# Interplay Between Exercise and Gut Microbiome in the Context of Human Health and Performance

**DOI:** 10.3389/fnut.2021.637010

**Published:** 2021-06-10

**Authors:** Matthieu Clauss, Philippe Gérard, Alexis Mosca, Marion Leclerc

**Affiliations:** ^1^Université Paris-Saclay, INRAE, AgroParisTech, MICALIS Institute, Jouy-en-Josas, France; ^2^Department of Physical Performance, Norwegian School of Sport Sciences, Oslo, Norway; ^3^Hôpital Robert Debré, Assistance Publique-Hôpitaux de Paris, Paris, France; ^4^Institut National de la Santé et de la Recherche Médicale et Université Paris Diderot, Sorbonne Paris-Cité, United Medical Resources 1149 Labex Inflamex, Paris, France

**Keywords:** inflammation, gut microbial ecosystem, gut microbial diversity, probiotics, sportomics

## Abstract

Gut microbiota and exercise have recently been shown to be interconnected. Both moderate and intense exercise are typically part of the training regimen of endurance athletes, but they exert different effects on health. Moderate exercise has positive effects on the health of average athletes, such as a reduction in inflammation and intestinal permeability and an improvement in body composition. It also induces positive changes in the gut microbiota composition and in the microbial metabolites produced in the gastrointestinal tract. Conversely, intense exercise can increase gastrointestinal epithelial wall permeability and diminish gut mucus thickness, potentially enabling pathogens to enter the bloodstream. This, in turn, may contribute to the increase in inflammation levels. However, elite athletes seem to have a higher gut microbial diversity, shifted toward bacterial species involved in amino acid biosynthesis and carbohydrate/fiber metabolism, consequently producing key metabolites such as short-chain fatty acids. Moreover, rodent studies have highlighted a bidirectional relationship, with exercise impacting the gut microbiota composition while the microbiota may influence performance. The present review focuses on gut microbiota and endurance sports and how this interconnection depends upon exercise intensity and training. After pointing out the limits of the studies so far available, we suggest that taking into account the microbiota composition and its metabolic contribution to human host health could help in monitoring and modulating athletes' health and performance. Such an integrated approach should help in the design of microbiome-based solutions for health or performance.

## Key Points

Moderate endurance exercise reduces inflammation, improves body composition and leads to positive effects on gut microbial diversity and composition and its metabolic contribution to human health. Endurance exercise exhibits positive effects on human health and on the gut microbial ecosystem, provided that the exercise intensity is controlled.

Elite athletes seem to have a higher gut microbial diversity and a shift toward bacterial species involved in specific pathways such as the production of short-chain fatty acids (butyrate, propionate).

Rodent studies suggest that the gut microbiota may influence performance.

Confounding factors such as diet, body composition, study design, and analytical methods limit the conclusions of the existing studies.

The balance between training load, performance, microbiota composition and functions should be monitored over time in a more integrated manner to optimize performance, health, and well-being and limit digestive diseases/issues in recreational as well as elite athletes.

## Scope of This Review

This review will focus on the interconnection between gut microbiota and exercise. Confounding factors such as diet can impact this interconnection. These factors will also be discussed in this review. Athlete cohorts, diseased populations and overweight populations will be used to expand on the effects and mechanisms of this interconnection. Specific animal models will also be highlighted to provide details on the mechanisms not yet clarified in humans.

## Endurance Exercise

In endurance exercise, a common definition of performance is the time to complete a certain distance. Therefore, athletes try to maximize their average speed during the defined distance to complete, but performance is always constrained by human body limits. In endurance exercises, researchers have been trying, for many years, to pinpoint the factors limiting performance from a physiological perspective and ways to overcome them.

First, during endurance aerobic exercise, muscles rely mainly on the breakdown of stored glycogen-glucose for energy production. However, as glycogen stores are limited, the existence of other energy sources is essential (1). These energy sources can rely on endogenous and exogenous substrates. Therefore, the intake of carbohydrates during exercise has been a widespread strategy to improve performance. Carbohydrates are absorbed in the blood flow due to transporters in the intestine. This step is crucial and often limiting in terms of performance (2), and training the gut to absorb exogenous energy substrates during exercise can improve endurance performance as well as provide a better experience for athletes (3).

Second, performance in endurance exercises is limited by the cardiovascular capacity, often measured using VO_2max_ (maximum oxygen uptake) - the maximum rate of oxygen consumption that the body can use during exercise. When a person trains at progressively higher intensities, oxygen uptake increases linearly to meet the demand of active skeletal muscles, until maximum oxygen uptake is reached (4). The principal limitation of the cardiovascular capacity is cardiac output. Cardiac output is defined as “the product of stroke volume and heart rate.” Cardiac output generally increases linearly with exercise intensity. This increase in blood flow can have major consequences for the digestive system including ischemia in the gut due to blood flow redistribution. This can lead to lower gastrointestinal (GI) disorders (abdominal pain or discomfort, bloating, diarrhea, constipation) as well as upper gastrointestinal disorders (stomach pain, nausea, vomiting) (5). The alteration of gut transit time is also detrimental to the microbiome balance. During long-lasting endurance exercise, such disorders have a high prevalence (6): 17% of subjects reported upper GI complaints in a 67-km race, and up to 47% reported upper GI complaints in a 160-km (100 Mile) race (7). Unsurprisingly, this is one of the main reasons why ultrarunners do not finish an ultramarathon (8).

In view of these elements, the proper functioning of the digestive tract and the associated microbiota need to be considered in order to perform well in endurance sports. The main focus of this review will therefore be the relationship between exercise and the gut microbiota in endurance sports.

## Gut Microbiota and Health Status

The human body is inhabited by a large number of bacteria, viruses, archaea and unicellular eukaryotes (9) called the microbiota (10). After a first estimate that the human microbiota contains up to 10^13^-10^14^ bacterial cells, 10 times more than cells in the human body (11), a recent update established a 1:1 ratio between the bacterial cells and the human body cells (12). Microorganisms are also widespread on the surface of the human body, colonizing the skin as well as the genitourinary, gastrointestinal, and respiratory tracts (10, 13). The gut microbiota is the focus of this review, and it is estimated that over 70% of all microbes in the human body are contained in the gut microbiota (11).

The microbiota refers to the assembly of microorganisms, while the microbiome is a larger term and not only refers to these microorganisms but also encompass their theater of activity, which results in the formation of specific ecological niches (structural elements, metabolites/signal molecules, and the surrounding environmental conditions) (14).

The gastrointestinal tract is an organ system that has many functions: it takes in food, digests it to extract and absorb energy and nutrients, and expels then the remaining waste as feces. It consists of the upper gastrointestinal tract formed by the esophagus and stomach and the lower gastrointestinal tract composed of the small intestine (duodenum, jejunum, and ileum) and large intestine (cecum, colon, rectum, and anal canal). The intestine has a large exchange surface area of ~80 m^2^ (15), due to the villi in the epithelium layer. The gut microbiota is located in the intestinal lumen, next to but also within the first outer layer of the mucus bilayer (16–18).

At the level of bacterial strains, as seen in classical microbiology, the gut microbiota demonstrates tremendous diversity and variation between individuals (19, 20). The human gut microbiota consists of four main phyla: Firmicutes and Bacteroidetes, quantitatively the most abundant, as well as Actinobacteria and Proteobacteria (21). The microbial populations can be stratified into 3 enterotypes and these bacterial gene correlation networks were shown to be driven by the following genera: *Prevotella, Bacteroides*, and *Ruminococcus* (21). Their relative prevalence has been shown to be largely driven by dietary habits (21, 22). The need to stratify into enterotypes is particularly relevant in clinical settings: for ranging from direct disease associations to prospective study stratification or even personalized dietary interventions or other gut modulation treatments (23).

The gut microbiota has coevolved with the host over thousands of years to form an intricate and mutually beneficial relationship (24). The microbiota offers many benefits to the host through a range of physiological functions affecting host nutrition, metabolic function, and maturation of the immune system (25, 26).

The gut microbiome contributes to digestion and promotes food absorption for host energy production (27). Microbiome fermentation leads to metabolites that are very relevant to athletes, such as short-chain fatty acids (SCFAs), lactate and branched-chain fatty acids. SCFAs are volatile products of microbial fermentation of undigested food, mainly fibers and, to a lesser extent, undigested peptides and amino acids in the large intestine, and can supply ~10% of the total energy for the host (25). The most abundant SCFAs are found at proportions of 60:20:20 for acetic acid (C_2_), propionic acid (C_3_) and butyric acid (C_4_) (26). SCFAs have distinct physiological effects: they can be used as energy sources by host cells and the intestinal microbiota, but they can also contribute to shaping the gut environment, influencing the physiology of the colon, and participating in different host-signaling mechanisms (27), as well as possessing some anti-inflammatory effects. SCFAs appear to be of paramount importance as a marker of changes in intestinal ecology (28) and highlight the close link between diet, the gut microbiota and metabolic function.

Secondary bile acids, produced in the colon by the microbiota, also exert effects on the metabolic function of the host, particularly on the metabolism of triglycerides and glucose (28, 29). Indeed, after being produced in the colon, they can be transported in the blood and reach a variety of organs, including the liver and kidneys.

The gut microbiota is highly linked to the host immune system (30, 31): protection from pathogens with the mucosal firewall, induction of effector T and B cell responses against pathogens, competition for nutrients with pathogens, production of antimicrobial molecules and metabolites that affect the survival and virulence of these pathogens, and reinforcement of tight junctions. It also helps in the stimulation and maturation of epithelial cells (32).

Another aspect of gut health is the interrelation among the gut microbiota, intestinal permeability and inflammation. For a recent review discussing the definition of a healthy microbiome see Shanahan et al. (33). Intestinal permeability describes “the control of material passing from the gastrointestinal tract lumen through the cells lining the gut wall into the blood circulation” (34). Transepithelial or transcellular permeability consists of the specific transport of solutes, thanks to specialized transporters, across epithelial cells. Paracellular permeability depends on transport through the spaces that exist between epithelial cells. It is mediated by the intestinal epithelium and regulated by intercellular tight junctions. This is the main route of the passive flow of water and solutes across the intestinal epithelium. Normally, permeability allows the maintenance of a balance between nutrients passing through the gut while keeping potentially harmful substances, such as antigens, from migrating to other body parts or fluid bodies (35). A disruption in gut mucus thickness (35), an imbalance in the gut microbiota composition or a decrease in gastrointestinal blood flow (34), caused by intense exercise, can lead to impairments in these fluxes. Therefore, harmful substances such as endotoxins from the outer membrane of Gram-negative bacterial strains, namely, lipopolysaccharides (LPS), can then pass through the barrier (36). Often, the LPS blood concentration increases together with inflammatory cytokines. Hence, chronic inflammatory responses can be established in the body with major consequences on host health. Moreover, alterations in gut microbiota have been linked to functional and inflammatory disorders (37).

## Assessing the Composition and Functions of the Microbiome: From Anaerobic Microbiology to Omics Methods

The composition and functions of the human gut microbiome can currently be assessed by several different and complementary techniques, from cultivation to a combination of “omics” techniques [(38), Table 1]. It is key to understand their strengths and limits to understand the data they provide and how to interpret them. In an increasing number of studies, different methods are being combined to obtain a better picture of the physiological impact of the microbiota, instead of only inferring functions from the bacterial composition.

**Table 1:** Analytical methods to study the microbiome [adapted from Lepage et al. (38), with permission].

**Method's name**	**Molecule**	**Use**	**Basic overview**
Phylogeny	16S rRNA, ITS	Determine bacterial composition and diversity	Who is there?
Metagenomics	Chromosomal genomic DNA	Determine gene contents from uncultivated microbes	What are they able to do?
Metatranscriptomics	Messenger RNA/cDNA	Determine microbial gene expression	What they actually do?
Metaproteomics	Proteins/peptides	Determine microbial proteins production	What is produced?
Metabolomics	Metabolites	Determine microbial and host metabolic profiling	Which molecules are there?

Non-targeted metabolomics approaches using nuclear magnetic resonance (NMR) have been performed on gut samples and body fluids from humans and animals. Among the hundreds of molecules detected, it has been estimated that between 15 and 20% of the metabolites measured derive from microbial metabolism (39). Hence, serum and urine measurements can allow us to understand the interplay between the microbiota and the human body/host.

## Relationships Between Exercise, Human Microbiota and Health

**Figure 1:**
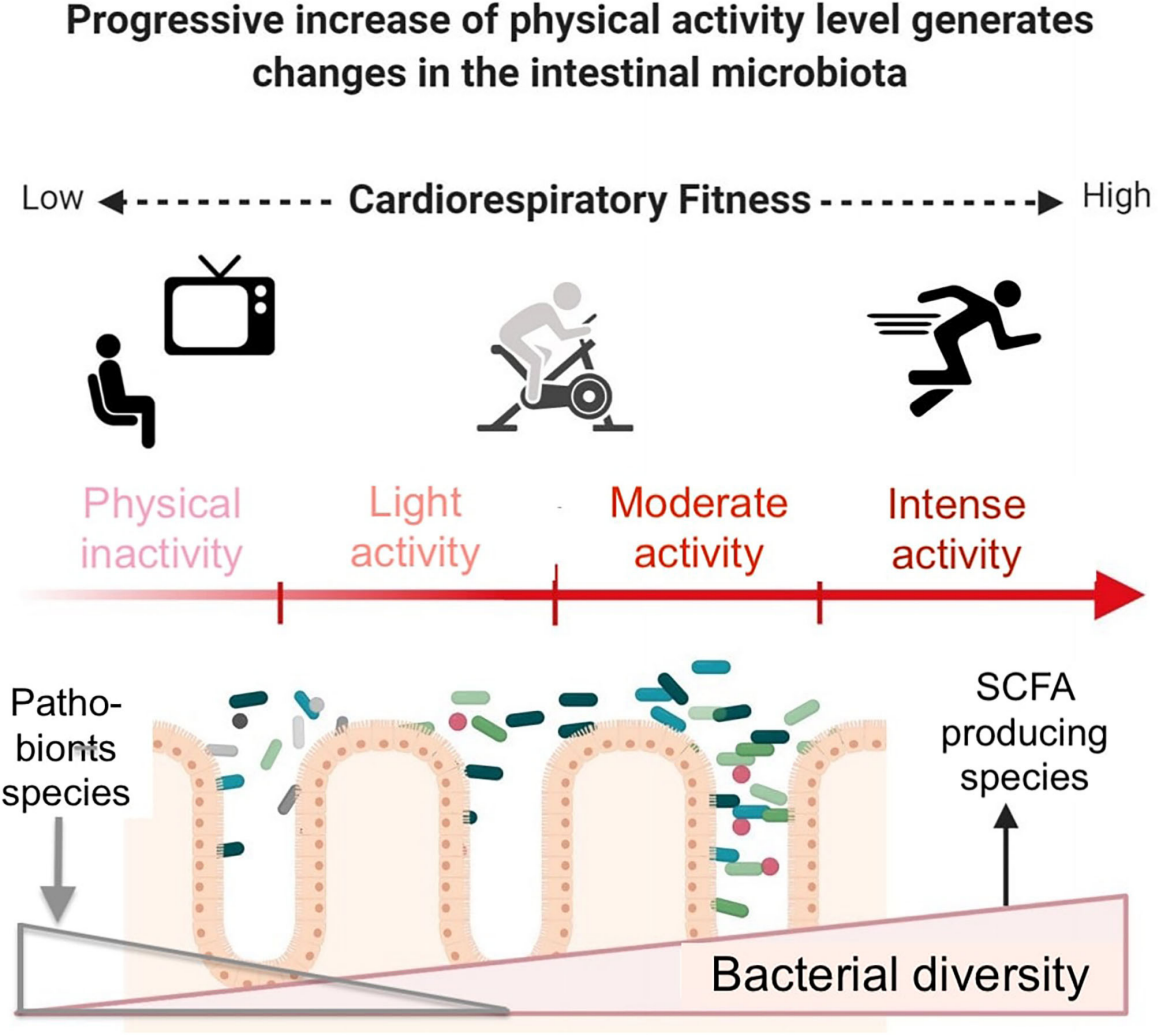
Beneficial effects of exercise and gut microbiota modifications in inactive subjects. Exercise induces beneficial molecular adaptations allowing the enhancement of cardiorespiratory fitness. Bacterial diversity increases, including SCFA- producing species. Conversely, pathobionts such as *E. coli* or *E. faecalis*, potentially disease-causing species which, under normal circumstances, are found as a non-harming symbiont, decrease. Longitudinal studies monitoring exercise intensity and modality, diet, subjects' characteristics and gut microbiota are still lacking. Modified from Aya et al. (40), with permission.

In endurance sports, both an acute bout of exercise and a long training period can have an effect on microbiota and health. Acute bouts of exercise can be separated into moderate and intense exercise. In the following section, intense exercise will refer to all physical exercises performed at a high intensity (>70% VO_2max_). Moderate exercise will refer to all other types of exercise (<70% VO_2max_). This review will include data on a wide range of participants: from overweight or diabetic subjects to elite athletes. This wide range of participants will make it possible to compare the different responses observed and to discuss the presence or absence of a continuum between all these populations (Figure 1) (40).

### Effect of An Acute Exercise Session on Human Microbiota and Health

One acute session of exercise at moderate intensity (<70% VO_2max_) leads to several health benefits. Some of these beneficial effects of moderate exercise on the host might be mediated by decreased intestinal permeability (41), which prevents pathogens from crossing the intestinal barrier and then reduces systemic inflammation. In parallel, an acute session of exercise at moderate intensity leads to several effects on the microbiota. The effect on the microbiota can be assessed by measuring the diversity or functions. α-Diversity represents the overall diversity of samples, while β-diversity compares how different bacterial species are distributed among different samples (42).

An investigation of the gut microbiota response to a half-marathon in amateur runners showed that the abundance of 7 taxa decreased, while the abundance of 20 bacterial clades increased (43). At the genus level, the top 4 biomarkers increased after the race were *Pseudobutyrivibrio, Coprococcus 2, Collinsella*, and *Mitsuokella* while *Bacteroides coprophilus* was the most decreased bacterial clade. Regrettably, no dietary questionnaire and no Bristol score that would indicate any gastrointestinal discomfort or bowel transit time difference were performed during this study. When omics methods were used, such as shotgun metagenomics and metabolomics, modest changes in gut microbial gene composition and functions were reported following increased physical activity (44). These data from two studies indicate that exercise can modify the gut microbial composition and production of SCFAs and thus fecal metabolites produced in the gut environment.

Intense (>70% VO_2max_) exercise sessions also exert beneficial effects on health. Based on the available studies, these sessions, compared to moderate exercise, seem to cause more significant disturbances than moderate exercise on the human body's homeostasis. Elite athletes have been shown to experience high levels of inflammation following an acute bout of exercise (45, 46) but also after intense exercise as attested by an increase in blood and urine markers of inflammation (47). However, elite rugby players have a lower inflammatory status compared to that of controls [higher interleukin-10 (IL-10) and IL-8; lower IL-6, tumor necrosis factor alpha (TNF-α), and IL-1β] (48).

Endurance athletes are particularly concerned with gastrointestinal symptoms. A study conducted during a long-distance triathlon concluded that LPS do enter the circulation after ultraendurance exercise. LPS may thus, with muscle damage, be responsible for the increased cytokine response and hence gastrointestinal complaints in these athletes (49). After this race, mild endotoxemia (ranging from 5 to 15 pg·mL^−1^; endotoxemia is present when LPS concentrations are >5.0 pg·mL^−1^) was evident in 68% of the athletes. In parallel, a 27-fold increase in IL-6 production was observed immediately after the race. Even if there was no significant correlation between LPS and IL-6 concentrations, these results indicate that increased intestinal permeability could occur simultaneously with an increased cytokine response and thus could contribute to an increased inflammatory response after exercise. Similarly, in a multiple-stressor military training environment, regardless of the diet group, both intestinal permeability and inflammation increased (50). Interestingly, 84% of the variability observed in the intestinal permeability changes could be accounted for by the *Actinobacteria* relative abundance before the training regimen together with changes in serum IL-6 and stool cysteine concentrations. Small intestine permeability was also increased during exertional heat stress (51). However, this increase was smaller in the glucose- or energy-matched whey protein hydrolysate groups than in the water-consuming control group. These changes, although negatively impacting host health, are only temporary and the benefits of such a high exercise load outweigh the temporary drawbacks.

After a multiple-stressor military training environment consisting of a 4-day cross-country ski hike, the subjects' microbiota showed an increased α-diversity and changes in the relative abundance of more than 50% of identified genera (50). Interestingly, the abundance of less dominant taxa increased at the expense of the dominant *Bacteroides*. Furthermore, in a study focusing on four well-trained male athletes performing a high-intensity unsupported 33-day, 5,000-km transoceanic rowing race, changes in microbial diversity, abundance and metabolic capacity (measured using 16S rDNA, metagenomics and metaproteomics, respectively) were recorded (52); microbial diversity increased throughout the ultraendurance event together with an increased abundance of butyrate-producing species as well as others associated with improved metabolic health and insulin sensitivity. The microbial genes involved in specific amino and fatty acid biosynthesis were also overrepresented. Notably, many of these adaptations in microbial community structure and function persisted at the 3-month follow-up. Microbial diversity thus increased even during intense exercise.

### Effect of Training/Fitness Status on Human Microbiota and Health

Beyond the effect of exercise load, the fitness status also impacts the microbiome. Regarding the relative importance of these two stimuli, the current consensus is that it is fitness that matters. This section will thus explain the effects of training/fitness status on human microbiota and health.

The microbiome of fit individuals, in good physical shape, has been shown to display increased butyrate production due to the increased abundances of key butyrate-producing bacterial taxa belonging to the Firmicutes phylum (*Clostridiales, Roseburia, Lachnospiraceae*, and *Erysipelotrichaceae*) (53). Similarly, the fitness measured by VO_2max_ was significantly correlated with the Firmicutes/Bacteroidetes ratio (54). However, none of the fitness, nutritional intake, or anthropometric variables correlated with the broad Firmicutes to Bacteroidetes ratio. In a 6-week intervention of endurance exercise in lean adults, exercise induced alterations in the gut microbiota composition and increased fecal concentrations of SCFAs in participants.

Cardiorespiratory fitness seems to be related to the relative composition of the gut microbiota in humans. When healthy elderly women were allocated to two groups receiving exercise interventions, either trunk muscle training or aerobic exercise training including brisk walking (55), the relative abundance of intestinal *Bacteroides* significantly increased in the aerobic exercise training group only. Interestingly, after stopping of exercise training, exercise-induced changes in the microbiota were largely reversed (56).

In another study in which women performed physical exercise to at least the degree recommended by the World Health Organization (“at least 150 min of moderate-intensity aerobic physical activity throughout the week, or at least 75 min of vigorous-intensity aerobic physical activity throughout the week”), exercise modified the composition of gut microbiota: eleven genera, measured by quantitative qPCR (quantitative real-time polymerase chain reaction), were significantly different between active and sedentary women. The former exhibited a higher abundance of the health-promoting bacterial species *Faecalibacterium prausnitzii, Roseburia hominis*, and *Akkermansia muciniphila* (57). In another 6-week endurance exercise study without dietary changes, metagenomic analysis (16S rRNA gene sequencing and Illumina metagenomic analyses) revealed taxonomic shifts, including an increase in *Akkermansia* and a decrease in *Proteobacteria* (58). Importantly, these changes were independent of age, weight, and fat percentage as well as energy and fiber intake. Similarly in male subjects with insulin resistance, both sprint intervals and moderate-intensity continuous trainings reduced systematic and intestinal inflammatory markers and increased Bacteroidetes phylum proportions (59).

**Figure 2:**
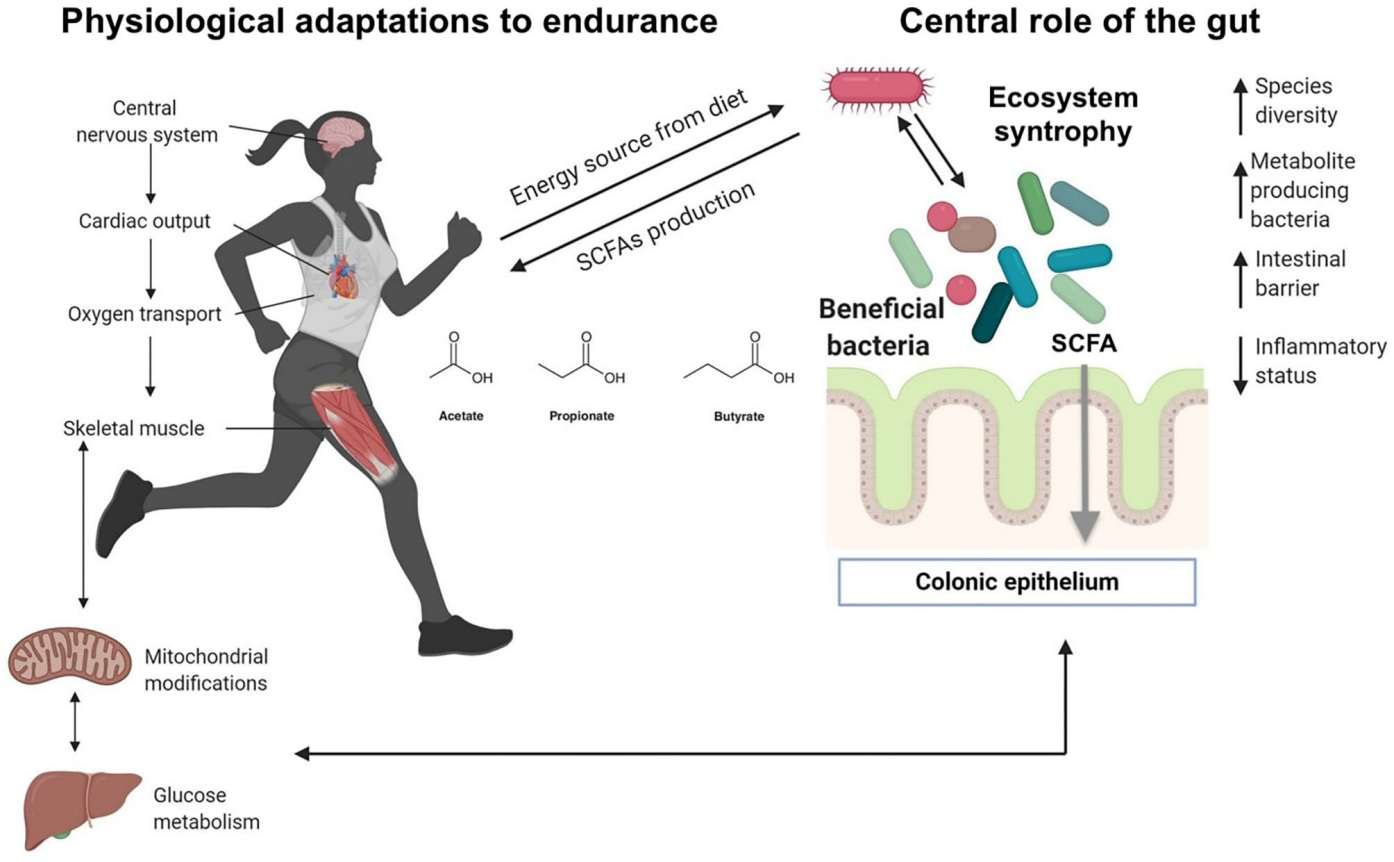
Ecosystem level adaptation of gut microbiota in athletes. Recent research indicates that unique gut microbiota may be present in elite athletes, and special and unique species can positively impact the host, providing metabolites from the fermentation of dietary fiber. Ecosystem level syntrophy: gut bacterial species can hydrolyze fibers and subsequently ferment the sugar monomers into SCFA, while other fermentative species depend upon the hydrolytic ones. Such a syntrophy have been described between *Bacteroides* and *Bifidobacterium* strains. Modified from Aya et al. (40), with permission.

The links between adaptations to endurance exercise and the gut microbiota are summarized in Figure 2. These conclusions need to be confirmed by longitudinal studies, but very few are currently available. One of them follows two initially unfit volunteers during 6 months while undertaking progressive exercise training (60). During this training period, fitness and body composition improved. In parallel, α-diversity increased as well as the concentration of some physiologically-relevant metabolites.

Elite athletes can also be used as a paradigm of the limit of the trained human body. After several years of intense training, elite athletes have special features in terms of athletic performance but also in terms of morphology and metabolic adaptations. A human study among elite rugby players vs. controls provided evidence of a beneficial impact of exercise on gut microbiota diversity: athletes had a higher diversity, representing 22 distinct phyla (48). However, the results indicated that these differences between the elite and control groups were associated with dietary extremes that could represent confounding factors.

In terms of the proportions of different bacterial populations and their inherent metabolic activities, a study conducted on elite rugby players demonstrated that athletes had relative increases in specific pathways (e.g., amino acid and antibiotic biosynthesis and carbohydrate metabolism) and fecal metabolites (e.g., microbial-produced SCFAs) (61). These pathways were associated with enhanced muscle turnover and overall health when compared with the control groups. Differences in fecal microbiota between athletes and sedentary controls showed larger differences at the metagenomic and metabolomic levels than at the compositional levels and provided added insight into the diet-exercise-gut microbiota paradigm. Another study in international level rugby players showed differences in the composition and functional capacity of the gut microbiome, as well as in microbial and human derived metabolites (62). The use of food frequency questionnaires reinforced the validity of these results. Focusing on cycling, another study compared professional and amateur athletes (63). At baseline, it was possible to split the gut microbiomes of the 33 cyclists into three taxonomic clusters: one with high *Prevotella*, one with high *Bacteroides* or one with a large set of genera including *Bacteroides, Prevotella, Eubacterium, Ruminococcus*, and *Akkermansia*. However, based on these taxonomic clusters, it was not possible to distinguish between professional or amateur cyclists. However, the high abundance of *Prevotella* (≥2.5%) significantly correlated with the reported weekly average exercise duration. *Methanobrevibacter smithii* transcripts abundance was also increased among a number of professional cyclists compared to amateur cyclists. A study in elite race walkers also reported that at baseline, the microbiota could be separated into the same distinct enterotypes with either a *Prevotella-* or *Bacteroides*-dominated enterotype (64). Moreover, intensive exercise combined with different nutritional strategies increased the relative abundance of *Bacteroides* and *Dorea* and reduced *Faecalibacterium* in the case of a low-carbohydrate/high-fat diet.

## Rodent Models: Reciprocal Effect of the Gut Microbiome, Exercise, and Performance

Rodent studies can be used to assess certain conditions that are difficult to test in human studies, particularly without use of overly invasive methods. Living conditions and diet are also easier to control in such studies. Rodent studies can help distinguish the effects of each of these factors distinctly.

Rodents are also good models for imitating human physiology. Indeed, in rodent studies, both the diversity and specific taxa of the gut microbiota have been shown to be impacted by exercise. However, it seems difficult to draw general conclusions at the moment due to many differences in the study designs (differences in diet, animal species/strain, and type of exercise). Indeed, while some studies found a reduction in Firmicutes and/or an increase in Bacteroidetes as a result of exercise (65–68), others showed the opposite effect (69–72), and others showed no effect (57, 58). Nonetheless, some bacteria generally appear to respond to exercise, including increased *Lactobacillus, Bifidobacterium*, and *Akkermansia* and decreased Proteobacteria. Finally, butyrate-producing taxa as well as SCFA production have been consistently shown to increase in response to exercise (61, 73), while the majority of studies also showed increased α-diversity following exercise.

Interestingly, some studies have investigated the effect of the gut microbiome on performance. The effect of the presence of the microbiome has been addressed by comparing germ-free (GF) to specific pathogen-free (SPF) mice and showing a higher exercise capacity in SPF mice (27). Moreover, exercise capacity improved in mice colonized with individual bacterial taxa compared to their GF counterparts. However, differences were observed between bacteria in the degree of impact (74). This suggests that if the gut microbiome may have a global positive impact on performance, its effect may depend on its composition. Interestingly, regardless of the bacterial species used to monocolonize GF mice, SPF mice always showed the greatest performance in a test of endurance swimming, suggesting that a more diverse microbiome may be necessary to exert beneficial effects. Recent studies have also shown that gut microbiota may be critical for optimal muscle function. Indeed, depletion of the microbiota using antibiotics led to a reduction in running capacity and in muscle contractile function (75, 76). Interestingly, similar results were obtained using a low-microbiota accessible carbohydrate diet that lowered SCFA production. Finally, restoration of the microbiota (75) or infusion of acetate (76) reversed the loss of endurance capacity and muscle contractile function.

An interesting aspect of animal studies is the possibility of performing fecal microbiota transplants (FMT). A few studies established that the beneficial health effect of exercise may be mediated through gut microbiome changes. Indeed, high-fat diet-fed mice receiving FMT from exercised donors not only showed markedly reduced food efficacy but also improved metabolic profiles (77). The transmissible beneficial effects of FMT were associated with the bacterial genera *Helicobacter* and *Odoribacter*, as well as an overrepresentation of oxidative phosphorylation and glycolysis genes in the metagenome. Similarly, it has been shown recently that the gut microbiome determines the efficacy of exercise for diabetes prevention. Exercise was first shown to improve glucose homeostasis only in a fraction of pre-diabetic individuals (responders). The microbiome of responders exhibited an enhanced capacity for the biosynthesis of SCFAs and catabolism of branched-chain amino acids. Moreover, the baseline microbiome signature could predict individual exercise responses. Remarkably, following FMT, gut microbiota from responders conferred the metabolic benefits of exercise to recipient mice (78).

Rodent studies have recently produced interesting new results, indicating that each exercise modality causes its own alterations of the gut microbiome (32). First, both voluntary wheel running and forced treadmill running altered many individual bacterial taxa, including *Turicibacter* spp., which had previously been associated with immune function and bowel disease. In mice fed a high-fat diet, exercise was proven to increase the Bacteroidetes phylum, while it decreased Firmicutes proportionately to the distance the mice ran (79). The high-fat diet component in this study is an important parameter to consider as it has been shown to cause modifications in mouse gut microbiota at nearly the same magnitude as exercise alone (80).

## Diet Can Modulate the Microbiome and Gut Health of Endurance Athletes

As in animal models, exercise and diet may together impact the composition of the human gut microbiota. For example, a study investigating the gut microbial response in amateur half-marathon runners observed some changes in 40 fecal metabolites and some shifts in specific gut bacterial populations. However, the authors concluded that these observed differences might have been the shared outcome of running and diet (43). As reviewed by Mitchell et al., the potential interactions between exercise, diet composition and their respective influences on the intestinal microbiome are not well-characterized (81).

In particular, the amount of fiber consumed should be taken into account before drawing any conclusions when comparing the results of different studies. Dietary fibers are defined as “carbohydrate polymers with three or more monomeric units, which are neither digested nor absorbed in the human small intestine” (82, 83) (Table 2). Their bulking effect on transit time, stool frequency, and gut health (84) comes from the fact that some fibers are not absorbed in the small intestine and are thus fermented in the large intestine. Consequently, differences in fiber consumption impact the type and amount of SCFAs produced by the microbiota (85). For example, the gut microbiota of children from Burkina Faso, whose diet contains a large amount of fibers compared to European children, was significantly enriched in Bacteroidetes and depleted in Firmicutes (86). Furthermore, significantly more SCFAs were found in Burkina Faso children's feces compared to in European children's feces. Species from the Bacteroidetes phylum mainly produce acetate and propionate, whereas butyrate-producing bacteria are found within the Firmicutes phylum (87). The increasing fiber consumption resulted in higher microbiota stability associated with higher microbiota richness.

**Table 2:** The different types of dietary fiber [modified from (83)].

**Types of fiber**	**Soluble or insoluble**	**Sources**	**Main bacterial genes for hydrolysis and known metabolomic products**
Cellulose, some hemicellulose	Insoluble	Naturally found in nuts, whole wheat, whole grains, bran, seeds, edible brown rice, and skins of produce.	Require multiple glycosyl hydrolases families^a^: GH2, GH5, GH8, GH9, GH44, GH48.
Inulin oligofructose	Soluble	Extracted from onions and byproducts of sugar production from beets or chicory root. Added to processed foods to boost fiber.	GH32 and GH91 releasing fructose fermented into SCFA. Increased butyrate in some studies.
Lignin	Insoluble	Found naturally in flax, rye, and some vegetables.	Short-chain fatty acids.
Mucilage, beta-glucans	Soluble	Naturally found in oats, oat bran, beans, peas, barley, flaxseed, berries, soybeans, bananas, oranges, apples, carrots.	Promote short chain fatty acids production through the EMP pathways od anaerobic digestion: SCFAs + CO_2_ + H_2_, CH_4_.
Pectin and gums	Soluble (some pectins can be insoluble)	Naturally found in fruits, berries, and seeds. Also extracted from citrus peel and other plants boost fiber in processed foods.	PL1, PL9 release galacturonic acid, fucose PL11 releases rhamnose. Increased propionate/acetate ratio in High Methoxy vs. Low Methoxy pectins
Polydextrose polyols	Soluble	Short length oligomers. Added to processed foods as a bulking agent and sugar substitute. Made from dextrose, sorbitol, and citric acid.	Fermented quickly in the ileum, producing H_2_, gas and bloating in some individuals.
Psyllium	Soluble	Extracted from rushed seeds or husks of plantago ovata plant. Used in supplements, fiber drinks, and added to foods.	Rich in arabinose and xylose. Increases water flow in the colon, used to treat constipation. Decrease sulfate reducing bacteria producing H_2_S. Increase butyrate acetate-dependent butyrate production?
Resistant starch and RS type 2	Soluble	In plant cell walls naturally found in unripened bananas, oatmeal, legumes. -RS type 2: In carbohydrates such a rice pasta, that were cooked then stored or refrigerated. Also extracted, acid-purified (RS4) and added to processed foods to boost fiber.	Starch, amylopectin cleaved by the GH13 gene family, releasing glucose. Mixed fermentation through the Embden-Meyerhof-Parnas pathway with acetate, propionate, butyrate.
Wheat dextrin	Soluble	Extracted from wheat starch, widely used to add fiber in processed foods.	Increases SCFA production differently according to the studies. Corn and potato dextrin also studied for SCFA increase.

a *Glycosyl Hydrolases description and classification in the CAZymes database. http://www.cazy.org/*.

*GH, glycosyl hydrolase; PL, pectin and pectate lyase; RS, resistant starch; SCFA, short chain fatty acids*.

Fiber intake is often low in the diet of athletes. Several studies, involving female artistic gymnastics, rhythmic gymnastics and ballet dance athletes (88), or competitive American adolescent swimmers (89) reported that athletes' fiber consumption was often below the nutritional guidelines of 25 g per day (based on a 2,000-calorie diet) (90). Only a few studies reported fiber consumption above the nutritional guidelines, and one of the few examples is female and male Dutch ultramarathon runners (91). Athletes may be reluctant to adopt such dietary habits because of higher satiety sensation or digestion and gastrointestinal discomfort issues (92). In parallel, to avoid gastrointestinal symptoms associated with exercise, some athletes turn to a low FODMAP (Fermentable Oligo-, Di-, Mono-saccharides And Polyols) diet to limit the presence of highly fermentable carbohydrates in their digestive tract (93). Indeed, undigested carbohydrates may increase the osmotic load in the small intestine and contribute to increased osmotic water translocation, volume, and physiological issues such as loose stool or diarrhea (94, 95).

Particular attention must also be paid when comparing elite athletes with sedentary controls. Indeed, dietary protein intake differs largely in elite athletes and sedentary controls diets (48). A recent study dealt with the effects of protein supplementation on the gut microbial composition (96). Protein supplementation increased the abundance of the Bacteroidetes phylum and decreased the presence of health-related taxa, including *Roseburia, Blautia*, and *Bifidobacterium longum*. The authors concluded that long-term protein supplementation may have a negative impact on gut microbiota. Likewise, a study comparing fecal microbiota characteristics among healthy sedentary men (as controls), bodybuilders, and distance runners found that daily protein intake negatively correlated with diversity in distance runners. This implies that a high quantity of protein in the diet may negatively impact the gut microbiota. Moreover, there was no difference in microbial diversity, but subject populations differed in terms of their gut microbial composition: *Faecalibacterium, Sutterella, Clostridium, Haemophilus*, and *Eisenbergiella* were the highest in bodybuilders, while *Bifidobacterium* and *Parasutterella* were the lowest. Some intestinal beneficial bacteria (*Bifidobacterium adolescentis* group, *Bifidobacterium longum* group, *Lactobacillus sakei* group, *Blautia wexlerae* and *Eubacterium hallii*) were the lowest in bodybuilders and the highest in controls. Thus, bodybuilders demonstrate a decrease in SCFA-producing commensal bacteria compared to controls (97).

## Probiotics as a Way to Impact the Gut Microbiome

Probiotics are defined as “a preparation of or a product containing viable, defined microorganisms in sufficient numbers, which alter the microbiota (by implantation or colonization) in a compartment of the host and by that exert beneficial health effects in this host” (98). Historically, probiotics have been used to mitigate intestinal issues linked to antibiotic treatment, travel, or illness (99). Until very recently, the beneficial effects demonstrated after probiotic consumption were immune modulation and strengthening of the gut mucosal barrier. The mechanisms included (1) modifications of gut microbial composition, (2) dietary protein modifications by the microbiota, (3) modification of bacterial enzyme capacity, (4) physical adherence to the intestinal mucosa that may outcompete a pathogen or inhibit its activation, and (5) influence on gut mucosal permeability (100, 101). There are also effects through interactions with immune intestinal cells or altering cytokine production, especially in the upper part of the gut, where probiotics may transiently dominate (102).

Compared to hundreds of commensal species inhabiting the human gut microbiota, probiotics are limited to specific bacterial strains, mostly within the genera *Lactobacillus, Bifidobacterium*, and *Saccharomyces* for yeasts, for regulatory reasons. *Lactobacillus acidophilus* and *Lactobacillus casei Shirota* have the longest history among known bacterial strains for application. In present-day commercial probiotic products, *Lactobacillus* spp. are well-represented, followed by *Bifidobacterium* spp. (103). There is today a high degree of consensus that the clinical effects of probiotics are strain-dependent, meaning that probiotic properties should be defined not only at the species level but also at the strain level (102).

**Figure 3:**
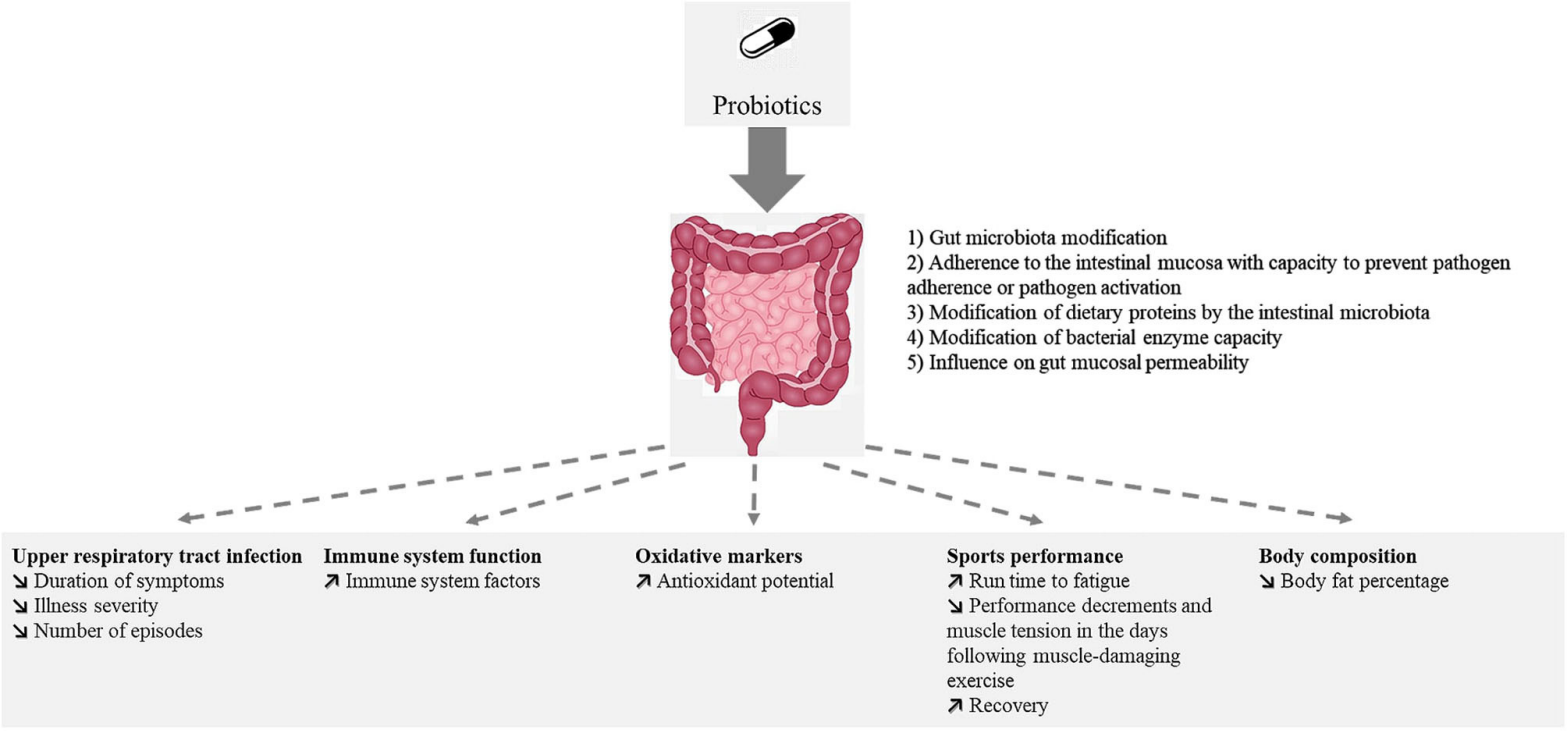
Reported effects of probiotic ingestion by athletes or subjects practicing moderate physical exercise.

Probiotics have been tested for different potential health effects on athletes. Figure 3 summarizes the reported effects of probiotic ingestion by athletes or subjects practicing moderate physical exercise.

The effects of probiotics on gastrointestinal symptoms and inflammation/oxidative have been studied in elite and competitive athletes. However, the effects differed between males and females, the latter group being less studied. Until recently, probiotic supplementation effects on sports performance have seldom been tested.

For example, *Lactobacillus rhamnosus* strain ATCC 53103, when tested in marathon runners, demonstrated no effect on the number of GI symptom episodes, but their duration was shorter in the probiotic group (104). In competitive cyclists, the number and duration of mild gastrointestinal symptoms were ~2-fold higher in the probiotic group (*Lactobacillus fermentum* PCC) (105). However, in males, there was a substantial reduction in the severity of gastrointestinal illness as the mean training load increased. Noticeably, the burden of lower respiratory illness symptoms decreased in males but increased in females. When sprint athletes consumed *Bifidobacterium bifidum*, their IgA, IgM, lymphocyte and monocyte percentages and CD4 counts were significantly higher than those of the control group (106).

*Lactobacillus helveticus* Lafti® L10 supplementation for 3 months in a population of elite athletes (triathletes, cyclists, and endurance athletes) showed, in the probiotic group, a decrease in the main markers of oxidative stress and antioxidative defense, such as malondialdehyde, advanced oxidation protein products and superoxide dismutase (107).

In male runners, multistrain probiotic supplementation (*Lactobacillus acidophilus, Lactobacillus rhamnosus, Lactobacillus casei, Lactobacillus plantarum, Lactobacillus fermentum, Bifidobacterium lactis, Bifidobacterium breve, Bifidobacterium bifidum*, and *Streptococcus thermophilus*) significantly increased running time to fatigue. In addition, probiotic supplementation led to small to moderate reductions in intestinal permeability and gastrointestinal discomfort (108).

In 24 recreational runners, probiotic supplementation for 28 days prior to a marathon race [*Lactobacillus acidophilus* (CUL60 and CUL21), *Bifidobacterium bifidum* (CUL20), and *Bifidobacterium animalis* subs p. lactis (CUL34)] was associated with a significantly lower incidence and severity of GI symptoms and limited decrease in average speed in the probiotics group compared to the control group (109). However, there were no significant differences in finish times between the groups.

Probiotic supplementation (*Streptococcus thermophilus* FP4 and *Bifidobacterium breve* BR03) was reported to likely enhance isometric average peak torque production, attenuating performance decrements and muscle tension in the days following a muscle-damaging exercise (110), where subjects performed 5 sets of 10 maximal eccentric contractions. In a similar study design, *Bacillus coagulans* GBI-30 6086, significantly increased recovery at 24 and 72 h and decreased soreness at 72 h post exercise (111). Probiotic supplementation correlated with a maintained performance and a small increase in creatine phosphokinase. Finally, *Bacillus subtilis* consumption during offseason training in female collegiate soccer and volleyball players, in conjunction with post-workout nutrition, had no effect on physical performance (112). However, body fat percentages were significantly lower in the probiotic group.

Altogether, these results show that probiotics may improve oxidative or inflammatory markers but have no proven effect on performance. Nonetheless, potential new generation probiotics, first identified in elite athletes' microbiome undergoing exercise, have recently shown promising results in mouse performance models (113). These bacteria belonging to the *Veillonella* genus feed on lactic acid and produce propionate, which may increase endurance capacity.

## Conclusion

In endurance sports, the effects of exercise on the microbiome depend upon exercise intensity and its duration. Training can also reinforce some of these effects or develop new effects. In return, changes in the gut microbiota diversity and composition can translate into a reduction in inflammation and gastrointestinal symptoms as well as the modification of hundreds of metabolites. Many of them are beneficial for the organism (SCFAs, secondary bile acids, etc.) and can allow endurance athletes to conduct huge volumes of training or to improve their sports performance. Probiotics can be used, in addition, to further potentiate these adaptations. However, research is still needed to identify the best bacterial strains and their methods of administration.

Some limitations of the studies presented are summarized below:

— As seen in many of the studies presented above, correlation does not mean causality. In addition, in a number of studies, it is very difficult to distinguish between the effects of exercise and diet on the gut microbiome variations. They could both act synergistically.
— Food intake: Most of the food questionnaires were not filled out by study participants, and the food frequency questionnaires validated for the general public are not fully adapted to sports and exercise. The different types of fiber, protein and supplements are usually not documented.
— 16S rDNA microbial data are often correlated to functions. However, the genome content of species with highly similar rDNA 16S sequences can differ. So, the correlation between 16S rDNA taxonomy and functions does have limits. Besides 16S rDNA, other methods should be used to decipher the functions of microorganisms of interest.

To overcome these limitations, Table 3 summarizes our main suggestions for future studies.

**Table 3:** Recommendations for more integrated studies in order to understand the interplay between exercise and gut microbiota in recreational athletes and elites.

A definition of common research protocols is necessary in order to standardize results and compare studies of different modes of exercise, different environmental conditions, different athletes' types.
**Longitudinal studies** are needed to understand the short-term effects of a training regimen but also the long-term microbial and physiological adaptations of regular exercise. For example, following athletes over 1 year would inform about switches in microbiota composition and functions during resting and performance training periods. This will then pave the way to personalized training protocols limiting exercise induced dysbiosis.
**Clinical studies** should be performed to evaluate the benefit of exercise on gut microbiota composition and function, as well as on health, in individuals at risk of specific diseases (e.g., diabetes, overweigh, cardiometabolic disorders, IBD, etc.). Animal models would validate the role of such microbiota exercise-induced alterations on health.
To supplement current data on the effect of exercise load, future studies should include endurance exercises of medium duration at moderate and high intensities. Such data can also be supplemented by long-term endurance exercises. If the nutritional aspects are controlled in these studies, this will allow us to identify the contributions of intensity as well as duration of exercise on changes in the intestinal microbiome.
**Common protocols for microbiome should be used**. Defined bio-informatics pipelines (microbial OTUs picking tools, metagenomes reads' mapping on existing datasets and on available bacterial genomes) and common statistical comparisons should be used in order to compare results between studies.
**Panels of metabolic energy and immune pathways in the host, and key metabolites for human health could be targeted**. Tools such as microarrays, qPCR for specific genes could be monitored at high throughput. Metabolites that are produced by the microbiota or by human cells can be monitored using the same mass spectrometry methods (NMR, LC-MS/MS, GC-MS).
**Food questionnaire**. Personalized applications regarding food intake are now available on smartphones and should be tailored toward athletes. The intake could then be correlated to blood and urine markers but also to microbiome composition.
The rationale for the choice of lower-fiber foods, as long as sufficient micronutrient status is ensured could be tested. Controlled tests including the microbiome could help determine which fiber or prebiotics, in which amount, can be better tolerated by athletes. The impact of the different types of fibers on the microbiota and the host depends on their chemical structure and on the microbial genes responsible for their hydrolysis (namely the Glycosyl Hydrolase genes). The GH genes panel should be analyzed from the microbial metagenomes. Breath tests should be performed, allowing to measure fermentation speed and choose the right fiber based on the individuals and sports modalities.
Protein intake should be controlled and ideally similar between the different tested conditions. Comparisons of different protein sources could be performed in a controlled manner. Then, metabolites that are biomarkers of microbial metabolism of specific amino-acids could be monitored (serotonin or spermidine from tryptophan, isovalerate produced from leucine, isobutyrate from valine).
**Studies should include the analysis of the microbiota composition and functions when evaluating the effects of different sport modalities** (training periodization, dietary supplementation, recovery techniques…). These data, in addition to those that are now generally measured, can help build a large model that can encompass many disciplines in order to develop a global vision of body adaptations through exercise.

*OTUs, Operational Taxonomic Units; qPCR, Quantitative Polymerase Chain Reaction; NMR, Nuclear Magnetic Resonance; LC-MS/MS, Liquid Chromatography Tandem Mass Spectrometry; GC-MS, Gas Chromatography-Mass Spectrometry*.

Some authors suggest applying a sportomics approach to “mimic the real challenges and conditions that are faced during sports training and competition.” Sportomics is defined as “non-hypothesis-driven research on an individual's metabolite changes during sports and exercise” (114). Furthermore, non-targeted analysis has opened “the door to a new era of high-throughput exercise-induced metabolic research” (115). Similarly, metatranscriptomics, metaproteomics and metabolomics microbiota analyses can help to (i) explain some of the sports-induced modifications and (ii) find new key targets to act on. We suggest adding longitudinal sportomics studies to microbiome monitoring through omics methods, together with dietary and well-being questionnaires.

Such an integrated approach opens the door to personalized nutrition/training program based on microbial composition. It could lead to microbiome-based solutions for health or performance by helping in the design of new supplements and also probiotics that would not necessarily be a unique strain but rather a consortium of species for a given metabolic outcome. In addition to new monitoring applications, this strategy could lead to optimized diets through personalized nutrition based on an individual's microbiome make-up and workout intensity.

## Author Contributions

MC wrote the first draft of the manuscript. ML coordinated the work. PG focused on animal models. AM on clinical context. MC, PG, AM, and ML revised the original manuscript. All authors approved the final manuscript.

## Conflict of Interest

The authors declare that the research was conducted in the absence of any commercial or financial relationships that could be construed as a potential conflict of interest.
